# Observations on Hematosis

**Published:** 1830

**Authors:** 


5. *Observations on Hematosis, with two cases in which this function was imperfectly performed.*—By SAMUEL JACKSON, M. D. Adjunct Professor of the Ins. and Prac. of Medicine, in the University of Pennsylvania.

Entertaining for the talents and acquirements of Dr. Jackson, feelings of the most profound respect, and still retaining a grateful recollection of his polite and communicative disposition towards the students of the Philadelphia Alms House, we cannot read, without deep regret, remarks like the following.

The secreted humours appear at this time to attract the entire attention of a large body of practitioners, whose sole object is to expel them from the bowels, and, like PURGON, *nettoyer les corps et en evacuer entierement les mauvaises humeurs* The whole practice of physic may be comprised, according to this system, in the universal prescription, with one alteration, of ARGAN, in the laughable satire of Moliere on the profession :—

*Calomelas donare,*
*Postea seignare,*
*Ensuita purgare,*
*Reseignare, repurgare, et recalomesare.*

Of infinitely more importance is it to appreciate and determine the state of the mucous surface of the primæ viæ and appendant organs, the immediate cause of the secretions, than to occupy the attention with the secretions themselves. These are much more harmless than is supposed. Nature finds but little difficulty in getting rid of them. The product of a certain condition of the secreting surface, they bear most generally a relation to that condition, whether it be natural or pathological, and when the two are in the same relationship, the secretions, however morbid, cannot react injuriously on the organs. But far otherwise is the case with the means employed to expel these morbid secretions. They are seldom in relation with the surface on which they act. Their action too is not in antagonism with the pathological state, but is of the same order—and it is not possible beforehand to pronounce positively that the therapeutic malady will supplant the pathological; that it will not act in the same line of march with the diseased action, and thus press it on in its destructive tendency. There are other means of equal potency, and of perfect safety, in changing the actions of the solids or organs, and thus effectually restoring the secretions to a healthy order. These for their simplicity, are despised by the PURGONS of medicine, who, ignorant of their mode of operation and of action, cannot conceive, that means which do not convulse the organs by their perturbating powers, can operate a salutary influence. This notion is of equal accuracy with that, which would attribute to nature no other remedy for the impurities of the atmosphere than a tornado or hurricane.

To what class of practitioners does the writer intend to apply these remarks? Where are these PURGONS to be found, who (to use the author's very classical expression) "*cannot conceive that means* which do not *convulse the organs by their perturbating powers* can *operate a salutary influence.*" Unqualified and unfounded abuse of this kind can serve, but to degrade the profession, and give some speciousness to the ranting of the miserable charlatans that are prowling in such numbers about the West, overspreading the land like the frogs of Egypt, and thrusting their disgusting faces into every man's bed chamber. The Doctor has assumed a principle which yet remains to be proved, viz: that this class of remedies is not "in antagonism with the pathological state, but is of the same order." There are many reasons for supposing that most purgative medicines, in their first impression upon the surface to which they are applied, are direct counterstimulants, and that they do not increase the peristaltic motion by stimulating the mucous surface. It is therefore entirely gratuitous to say that *the therapeutic malady supplants the pathological.* Gentlemen who deal largely in sarcasm should be very certain of the grounds upon which they stand.

In addition, we will simply remark that medicine, in common with the other branches of science, has long been burdened by modes of expression, drawn from absurd and unintelligible hypotheses, oppressive to the student and revolting to literary readers. With the progress of literature and science, good taste and more correct views have been gradually removing this evil ; but the progress of Medicine, for a variety of reasons, has been slower in this respect than that of her sister Sciences. There is no subject, upon which society in general speculate so much, and know so little, as upon the subject of medicine. The whole science is founded upon observation. The modus operandi of medicines, and the proximate causes of disease are so far involved in obscurity that physicians, when called upon for reasons, can only offer the results of experience. The inquisitive disposition of the public, however, cannot be satisfied without a rationale for every thing ; and hence, a strong temptation to mountebanks and innovators, to impose upon their curiosity by substituting obscure and unintelligible modes of expression for a frank avowal of the limited extent of professional attainment. Men of candor and correct views cannot stoop to this imposition, and therefore, frequently compete to great disadvantage, with men who are, in every respect, their inferiors.

It is no small discredit to the opinions of Broussais, that his objections to the established practice are founded upon *a priori* reasoning, and that to sustain these positions, his followers have been compelled to revive speculations and modes of expression which, the good taste and good sense of the profession have long since rendered obsolete ; and if his opinions are true, it is at least a subject of regret, that they are too often couched in terms which are intelligible only, to the ear of the initiated.

Medical writers will find the English language sufficiently copious, without the introduction of phrases which violate its idiom. We certainly think therefore, although with the utmost deference to the opinions of others, that such expressions as, "medicines in relation with the surfaces upon which they act;" "in antagonism with the pathological state;" "convulse the organs by their perturbating powers;" "operate a salutary influence," should be most carefully avoided; and especially by men whose writings are likely to be considered as models of imitation, by the younger members of the profession.

The object of the paper is to place before the public a malady of not unfrequent occurrence in Dr. Jackson's practice in which the prominent symptom is a sudden dimunition, amounting almost to a disappearance of the hematosine or colouring matter of the blood.

*Case* 1. Miss M'C——, aged 19, lymphatic temperament, hair light brown, eyes a light grey, complexion fair, full embonpoint, limbs, round and smooth, chest expanded.

The patient had heretofore enjoyed uniform good health.—In November 1829, having exerted herself much more than usual and taken very little rest for three nights she was very much overcome with fatigue. From this time she began to notice that slight exercise produced unusual fatigue and excited palpitations of the heart, and a throbbing obvious to the eye above the sternum and on the right side of the neck, with a sense of beating in the head and through the limbs.

As these symptoms increased so as to prevent all active exertion the Doctor was requested to visit her, and saw her on January 4th 1830. As she had been perfectly quiet nothing could be observed but slight throbbing above the sternum and right clavicle. The chest when examined by the stethescope gave healthy indications in every part, except that the impulse of the heart was rather louder and stronger than usual, but not to an extent that indicated any morbid or organic change. The appetite was excellent, and diet too full, digestion perfect, bowels regular, evacuations natural. The tongue was clean and moist, and as well the gums, lips and inside of the mouth, was without colour.

The fact of the patient not having sustained any loss of blood seemed to forbid the suspicion of anemia. Dr. J. was inclined to suspect aneurism. And a consulting physician having concurred in this opinion, VS. was directed on the 10th January with a reduced diet. All the symptoms having been aggravated by this course and the blood consisting almost entirely of serum, he came to the conclusion that the case was one of anemia, or what is nearly of the same nature, a deficiency of the fibrine and hematosine of the blood. Phosphas ferri gr. 5. sulph. quinæ ss. every four hours; diet more generous. Under this treatment the patient gradually recovered, the colour returned to the lips, tongue and cheeks, the complexion resumed its healthy aspect and by the 10th February her health was perfectly restored.

*Case* 2. Mrs. W——, aged twenty, of temperament very similar to the last, was married in the winter of 1828–29, her health since that time has been imperfect, though never sufficiently ill to require medical attendance. Became pregnant and was confined after an easy labor in the second week of February 1830. On the third day, she complained of an incessant noise in the head which entirely prevented sleep, alternating with pain, for which she was bled, leeched and cupped in the head, and the bowels freely evacuated by purgatives, without benefit.

February 21st. Dr. J. saw her in consultation. The alternation of the pain and distressing noise in the head still continues, hearing very acute, sleeplessness, skin of the natural temperature, of a death-like paleness; tongue large, moist and colourless; lips equally pale; pulse large, full but without strength; stomach composed, and bowels without tenderness; blood drawn for the relief of the head, presented a small, loose coagulum floating in a large quantity of serum. This having given no relief, blisters were applied to the ancles, and an anodyne portion administered every two hours. February 22d, Some sleep was procured, head less distressed; a mild tonic prescribed. Evening, solution sulp. morphiæ. 23d, Night had been very much disturbed, nervous agitation, delirium, pulse 160, head very hot, feet cold. Hair cut close, cold constantly applied ; warmth to the feet. This treatment produced some relief, but the unpleasant symptoms were again superinduced by a resort to mild stimulants, and the case became further complicated by epigastric distress, with thirst, diarrhea and inflammation of the vulva. A blister was applied to the epigastrium, fomentations to the vulva ; and as the mildest tonics, and opiates in their least objectionable form, deranged the stomach, all internal stimulants were abandoned, and the sulp. morphiæ and sulp. quinæ were applied to the blistered surfaces. The symptoms improved very gradually. On the 20th March phosphas ferri, grs. v. sulp. quinæ 1-4 gr. were prescribed four times a day, and on the 30th, the patient could walk a little with assistance, the lips had more colour but were still unnaturally pale. From this time she gradually recovered.

From these cases Dr. J. draws several important inferences. The strong disposition to inflammation, entirely prohibited the use of internal stimulants, at a time when the state of the vascular system entirely precluded all evacuating measures. The application of cold to the head, with the repulsive action of blisters, sinapisms and heat to the extremities, kept the cerebral excitement in check, and eventually subdued it. There was no intermission to the cold applications for ten days. They were so grateful to the patient that she would not suffer them to be discontinued. This case affords a striking instance of the occasional importance of the endermic application of medicine. The sulp. quinæ in the present instance, besides fulfilling its general indications, gave vigour to the capillary circulation, and thus served to control the internal irritation, or in the words of Dr. J. "constituted a powerful revulsive or controlling power, antagonizing the morbid irritations of the internal dermoid tissue or mucous membranes." This case further proves that redness is not essential to inflammation, and that the intensity of the colour is not in proportion to the activity of the inflammation. Hence when the colouring matter of the blood is deficient, as it frequently is in the highly lymphatic temperament, the colour of the inflamed surface after death will afford no indication of the extent of the previous morbid irritation. In this case no difference was to be observed between the colour of the sound and blistered skin.

Dr. J. thinks that the remedies principally indicated are, "the chalybeate and more permanent vegetable tonics, introduced into the organism in a manner not to interfere with the digestive organs, when in an irritated state. The tonic and diffusive excitement of the mild mercurial preparations, gradually introduced, to avoid a pathological disturbance, are also of great utility in restoring the healthy condition of the blood."

*American Journal of the Med. Sci.*—J. C. F.

